# Should Men Take Prenatal Vitamins?

**DOI:** 10.4172/2161-038X.1000139

**Published:** 2014-07-01

**Authors:** Jason M Scovell, Ranjith Ramasamy

**Affiliations:** Division of Male Reproductive Medicine and Surgery, Department of Urology, Baylor College of Medicine, Houston, TX, USA

## Short Communication

Congenital abnormalities are the leading cause of infant mortalities in the United States (25%) and across the world [1]. Public health interventions have included reducing maternal disease, prenatal care of mothers, reduction of exposure to teratogens (agent that can disturb the development of an embryo or fetus), and nutritional interventions [2]. Potentially the most significant nutritional intervention has been the prenatal supplementation of folate, which has been shown to reduce the incidence of neural tube defects [3], limb malformations, urogenital abnormalities, cardiovascular malformations [4], and cleft lip or palate [5].

The influence of paternal diet on congenital abnormalities and fertility is still being elucidated. A recent mouse study by Lambrot et al. furthered our understanding of the effects of paternal folate deficiency [6]. Mice were fed a Folate Deficient (FD) or Folate Sufficient (FS) diet, and were assessed for birth defects. Additionally, genome-wide and spermatic methylation studies were performed. Folate deficiency was associated with an increase in DNA damage in spermatocytes. However, there was no difference in sperm tail DNA fragmentation, tail length, or motility (p>0.05). Mice fed with a folate deficient diet were less fertile (52% vs. 85%) and experienced a greater post-implantation loss than mice fathered by mice with a folate sufficient diet. Gross anatomical abnormalities were greater in mice fathered by FD mice (27% vs. 3%) which included craniofacial, hydrocephalus, limb, and muscle/skeletal defects. Another study investigating the effect of a paternal folate-deficient diet in mice found a relationship with decreased placental weight, placental folate and an increased expression of the folate transporter folate receptor [7].

A link has been found between paternal dioxin exposure, a component of the herbicide Agent Orange used in the Vietnam War, and spermatazoidfolate deficiency resulting in an increased rate of spina bifida [8]. Although the precise mechanism is unclear, it is thought that epigenetic modifications (study of heritable changes in gene activity that are not caused by changes in the DNA sequence) play a role in both neural tube defects and decreased fertility of men with a folate deficiency [8]. However, a prospective study of 42 couples, who had paternal methotrxate exposure at the time of conception, did not identify any congenital abnormalities [9]. In the literature, 2 out of 23 children fathered by men on methotrexate were found to have congenital abnormalities. The role of paternal nutrition on epigenetically linked heritable diseases such as cardiovascular and obesity have been suggested in epidemiological studies [10].

Human studies examining the role of paternal micronutrient supplementation are scant. In 2008, Young et al. investigated the role of supplemental folate, zinc and antioxidants (vitamins C, E, and β-carotene) on spermatocyte aneuploidy. Men consuming the most folate (>75^th^ percentile) had a lower frequency of disomy 21, X, sex nullisomy, and lower overall sperm aneuploidy [11].

It is clear that a deficiency of paternal micronutrients could affect the development of offspring through epigenetic regulation. However, it is unclear at this time whether or not supplementation of vitamins such as folate can reduce congenital anomalies in men with an otherwise well-balanced diet. A prospective randomized control trial is necessary to adequately determine the role for male prenatal vitamins. Due to the lack of conclusive evidence, we cannot recommend the ubiquitous use of prenatal vitamins, in particular folate, in men attempting to plan a pregnancy with their female partner at this time.
